# Technical Note: Effects of Makeshift Storage in Different Liquors on Larvae of the Blowflies *Calliphora vicina* and *Lucilia sericata* (Diptera: Calliphoridae)

**DOI:** 10.3390/insects12040312

**Published:** 2021-04-01

**Authors:** Senta Niederegger

**Affiliations:** Department of Forensic Entomology, Institute of Legal Medicine at the University Hospital Jena, 07740 Jena, Germany; senta.niederegger@med.uni-jena.de

**Keywords:** forensic entomology, biological variation, death time estimation, alternative storage, casework

## Abstract

**Simple Summary:**

Sometimes, police need to collect fly maggots as evidence. If the proper equipment is not at hand, alternatives might need to be found. This evidence can later be given to a forensic entomologist for further examination. The alternative methods, however, can have unknown effects on the samples. We placed maggots in different alcoholic beverages and measured size changes happening over time to provide experts with such information. Our results show that low alcohol beverages can cause samples to shrink. With knowledge about these specific effects, the samples can still be used for casework in forensic entomology.

**Abstract:**

Unexpected findings of forensically important insects might prompt makeshift storage in alternative liquids if the proper equipment is lacking. The assessment of whether such evidence can still be used and correctly interpreted can be difficult. In this study, the effects of using alcoholic beverages as storing agents for post-feeding larvae of *Calliphora vicina* and *Lucilia sericata* were analyzed. Larvae were killed with boiling water (HWK) or placed alive into four alcoholic liquids: two spirits, vodka and brandy, and two liquors, Jägermeister and peppermint schnapps. Storage effects were documented after one day, nine days, and one month and compared to larvae treated according to guidelines for forensic entomology. Results show that the method of killing larvae is more important than the storing medium. Storage of HWK larvae in high-alcohol/low-sugar spirits had almost negligible effects on both species, while all fresh larvae shrank significantly. High sugar contents of the beverages might additionally lead to shrinkage of larvae.

## 1. Introduction

Forensically relevant flies deposit their eggs on cadavers and carrion where their offspring hatch, feed, and develop [1,2]. This process was described and even denominated as a “biological clock” for the estimation of the minimal postmortem interval (mPMI) [3,4].

Oftentimes, human bodies colonized by fly larvae are discovered within a domestic setting. In many instances, investigators might presume natural death. Police personnel, as a precaution, might still want to collect and preserve a number of larvae. Circumstances indicating a crime might become known only after the body was cremated and the apartment cleaned. It can take an unpredictable amount of time for a previously unsuspicious death to change into the result of a crime.

The guidelines of forensic entomology [5], which recommend using boiling water for killing and fixation and ethanol for storage of fly larvae, should always be followed when collecting and storing insect evidence. The best medium for storage in forensic entomology casework is 70–80% ethanol [6], while some even suggest 70–95% ethanol [7]. The effect of numerous other media of known fly larvae killing and preservation methods have been investigated, from formaldehyde [6,8] to Kahle’s solution [8] and San Veino [9]. Investigators, however, might not always be able to provide ethanol, other chemicals, or hot water when larvae are unexpectedly found at a scene. In such cases, imaginative caseworkers might turn to materials at hand for emergency storage of larvae. Oftentimes, a range of alcoholic spirits or liquors are present right at the scene or a liquor store is nearby.

The aim of this study was to evaluate whether such evidence can still be interpreted and the results presented in court. To achieve this, post-feeding larvae of two species were treated as recommended in the guidelines, but stored in easily achievable alcoholic liquids, as well as 70% ethanol. Furthermore, living larvae were killed by transferring them directly into the tested liquors. Effects of storage up to one month were investigated.

## 2. Materials and Methods

Larvae of two forensically important fly species were used in this study: *Calliphora vicina* Robineau-Desvoidy, 1830 and *Lucilia sericata* (Meigen, 1826) from laboratory colonies kept in a climatic chamber at 21 °C with 60% humidity. Larval age was synchronized by 24 h periods, during which flies were offered minced meat for oviposition. Emerging larvae were left undisturbed in a rearing container for six days until most had reached peak length and started migration.

The larvae were extracted from the rearing container and randomly assigned to two treatments and five storing liquids. Fresh larvae were transferred directly into their assigned liquids. HWK (hot-water-killed) larvae were killed by placing not more than 20 individuals at a time in a tea strainer and doused with boiling water for at least 20 s; after dabbing with a paper towel, they were transferred to their assigned liquids. The abbreviation HWK (hot-water-killed) was established for this method of killing before [6] and maintained even though boiling and not just hot water was used.

To investigate the effect of alcoholic beverages as storage agents, popular spirits like vodka (Kaliskaya, 37.5% vol.) and brandy (Chantré, 36% vol.), as well as liquor specialties like Jägermeister (35% vol.), a well-known brand of herbal liquor as representative for locally diverse brands, and peppermint schnapps (18% vol.) due to its low alcohol content, were used. Fresh larvae were also stored in 70% ethanol. A second trial was performed five days later, for a total of 350 larvae (Table 1).

HWK post-feeding larvae of *C. vicina* and *L. sericata* stored in 70% ethanol were used as control and their lengths at the respective time served as reference points for both HWK and fresh larvae.

For each measurement, larvae were extracted from their vials, placed into a petri dish, photographed with a camera and a Zeiss Stereomicroscope Stemi 2000, and put back into their original vials without changing the storing medium. Length of the larvae was measured using Zeiss software in the images yielded from the microscope and documented.

In the first trial, maggots were measured after 2 h, but because at least 25% of the fresh larvae were still alive after this time, the data were disregarded and the measurement was not repeated. Subsequent length measurements were performed after 1 day, 9 days, and 1 month. These periods were deemed reasonable for domestic deaths to potentially change from unsuspicious to ominous.

A nonparametric Mann–Whitney U test was performed to compare samples of different sample sizes to their control group. Statistical analyses were conducted using IBM SPSS Statistics 26.

Storage temperature was not altered from laboratory average temperature of 20 °C, as a study of Richards et al. [10] showed no statistically significant effect of storage temperature between −25 °C and +24 °C in larval length and weight.

## 3. Results

**Figure 1:**
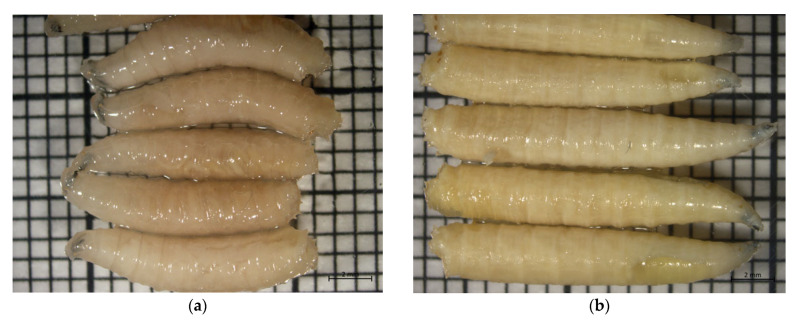
Larvae of *Calliphora vicina* after one day in 70% ethanol, (**a**) fresh: larvae placed into liquid while alive, (**b**) HWK: larvae hot-water-killed before placement in liquid.

When living fly larvae were placed directly into their respective storage liquids, all animals squirmed and contracted (Figure 1a). Larvae were still alive for up to three hours in their respective media. Killing with boiling water, on the other hand led to death and instant straightening of the whole body (Figure 1b). The first length measurements were performed after one day (Table 1).

**Figure 2:**
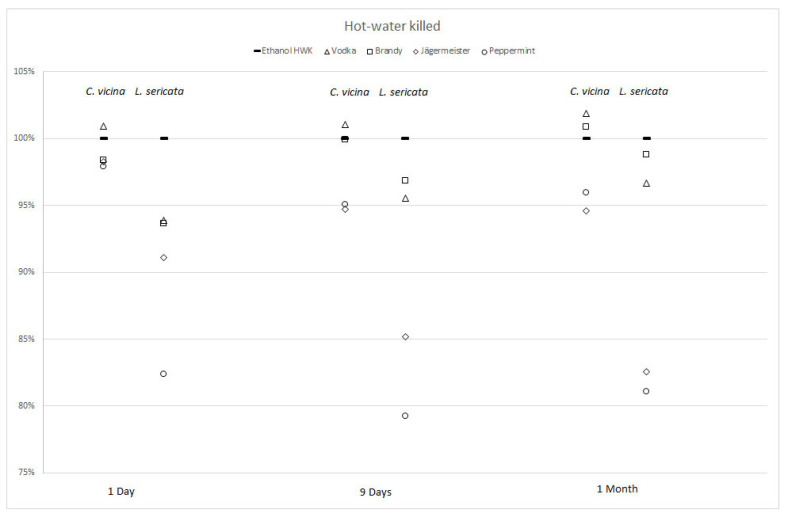
Percentage changes in lengths for *Calliphora vicina* (left column) and *Lucilia sericata* (right column) post-feeding larvae after hot-water-killing (HWK) and storage in four alcoholic liquids for 1 day, 9 days, and 1 month compared to HWK larvae in 70% ethanol (=100%).

The first experiment compared lengths of HWK larvae of both species stored in alternative alcoholic liquids to their counterparts treated according to the guidelines for forensic entomology [5] in 70% ethanol (Figure 2 and Table 2).

After one day, sizes of all *C. vicina* larvae in all spirits and liquors were within two percent points compared to controls, and thus not significantly different. Larvae in peppermint schnapps over time remained shorter than the control, but in a statistically nonrelevant range. Samples in Jägermeister shrank most and resulted in significantly shorter larvae than the controls. Lengths of larvae in the two spirits, vodka and brandy, in contrast, slightly increased to more than 100% of the size of control larvae over time, but differences were not statistically significant (Table 2).

Larvae of *L. sericata* in alternative liquids were 6–18% smaller than their controls on day one, and thus significantly different. Over time, larvae stored in brandy expanded the most and their lengths reached 99% of the size of controls. Larvae in vodka reached 97% of the size of controls by the end of the storage time of one month. These differences were no longer statistically significant (Table 2). Sizes of larvae in Jägermeister and peppermint schnapps differed more from controls over time. Lengths of larvae in Jägermeister shrank from 91% on day 1 to 83% of the size of controls after one month. Larvae in peppermint schnapps shrank from 82% to 79% on day nine, but reversed to 81% by one month storage time (Figure 2).

**Figure 3:**
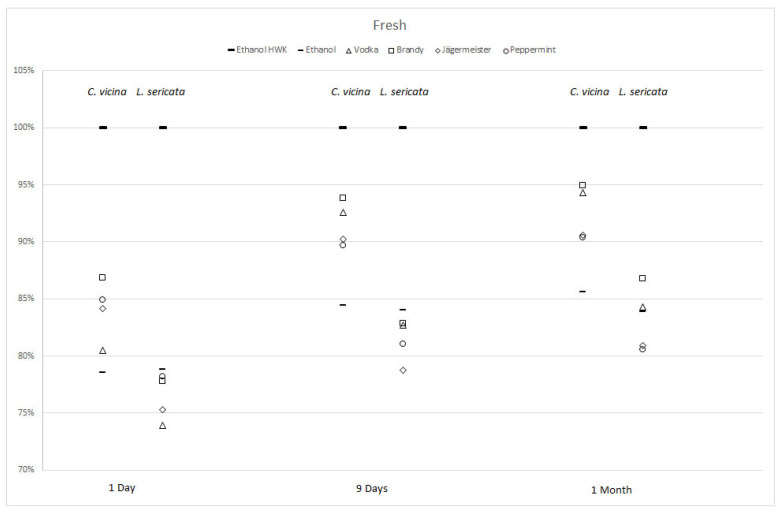
Percentage changes in lengths for *Calliphora vicina* (left columns) and *Lucilia sericata* (right columns) post-feeding larvae stored alive in five alcoholic liquids for 1 day, 9 days, and 1 month compared to hot-water-killed (HWK) larvae in 70% ethanol.

The second experiment compared fresh larvae stored in 70% ethanol and alternative liquids without previous hot-water-killing to the same control group of HWK larvae in 70% ethanol (Figure 3).

All freshly stored larvae were smaller than their HWK controls and all differences were statistically significant (Table 2). On day one, *C. vicina* larvae freshly stored in ethanol were 22% smaller, and fresh *L. sericata* larvae in ethanol were 21% smaller than their controls. These values represented the lowest discrepancy for *L. sericata*, while it was the highest recorded for *C. vicina*.

While the differences in sizes between fresh larvae of *L. sericata* and their control group were larger than in *C. vicina*, the range of variation between liquids tested was larger in *C. vicina*. Sizes of fresh larvae in all liquids over time approximated the sizes of control larvae for both species, but differences remained significantly different (Table 2).

## 4. Discussion

The effect of numerous methods of scientific killing and preservation for fly larvae have been investigated [1,6,7,8,9,10] to date. No investigations, however, have been conducted on the effects of improvised methods, such as the use of alcoholic beverages for storage of fly larvae, which might become necessary due to a lack of equipment.

For this study, post-feeding *C. vicina* and *L. sericata* aged six days were used. Numerous aspects concerning the larvae of these two forensically important species are well investigated [11,12,13,14,15,16,17,18,19,20,21,22,23,24,25,26,27,28,29,30,31,32,33,34,35,36,37,38,39,40,41,42,43,44]. At constant temperatures of about 20 °C, they develop at very similar rates. Days to maximum length, and thus the beginning of the nonfeeding stage, is given for *C. vicina* as 5.12 days and for *L. sericata* as 5.81 days [45]. This temperature regime, however, is marginally suboptimal for *L. sericata* [46,47], which resulted in slightly diminished oviposition, hatching activity, and sample sizes for this species (Table 1 and Table 2).

Adams and Hall [6] noted that the rate of expansion in larvae was highest during the first 3 h in 80% ethanol. In our study, at least 25% of the fresh larvae were still alive after two hours. Placement of living larvae into liquids led to contraction in all samples, which is due to the long drowning and suffocation process. Some larvae were still alive after three hours. The early measurement data could therefore not be incorporated. This resulted in a lack of data on the initial length of larvae tested. There is, to our knowledge, only one method to measure living larvae using a geometrical micrometer [48]. This method, however, is not suited for measurement of 350 mobile larvae in a reasonable amount of time.

The first measurements were performed the next day to ensure the death of all larvae. Subsequent measurements after nine days and one month were conducted to account for potential revelations of new information in casework. It takes at least a few days for a previously unsuspicious domestic death to change into a potential crime due to newly discovered evidence. It can also take up to a few weeks until the samples are sent to a forensic entomologist. Handling of samples was restricted to avoid damages.

Even though the age of larvae was synchronized by limiting oviposition for 24 h, the range in sizes was considerable (Table 1), with maximal sizes consistent with literature [10,34,46]. Biological variation in length within larvae of the same species was larger than the largest experimental difference in average lengths (Table 2). Size ranges are seldom specified in publications on development of forensically relevant insects. The provision of such data should be encouraged in publications on forensic entomology. It could contribute to the awareness of biological variation in entomological samples. Furthermore, analyses should not be based on larval sizes alone, but should incorporate the developmental stage in order to differentiate between an unusually large feeding larva and an unusually small post-feeding individual. Such tasks are especially difficult for nonexpert police personnel. It is therefore important to develop illustrated guidelines designed specifically for nonexperts in order to capacitate police personnel to perform useful insect collections.

The effects of alternative storage over all alcoholic beverages and both treatments were smaller in *C. vicina* than in *L. sericata (*Figure 2 and Table 2). This indicates that *C. vicina* might be a more robust fly species, which is also reflected in a wider temperature tolerance [47].

Comparison of HWK larvae showed that sizes of *C. vicina* larvae stored in the spirits vodka and brandy, as well as the liquor peppermint schnapps, were statistically similar to their controls in all measurements (Figure 2, Table 2). The effects of vodka and brandy were also statistically nonsignificant in HWK larvae of *L. sericata* after nine days and after one month. The European spirits regulation (EU Spirituosenverordnung [49]) defines vodka as a spirit gained from distillation of potatoes or grain. The maximal content of sugar allowed is 8 g per liter, alcohol content must be at least 37.5% vol. Brandy is a liquor produced by distilling wine and the sugar content allowed is no more than 35 g per liter, alcohol content must be at least 36% vol. Herbal liquors like Jägermeister and peppermint schnapps are required to contain at least 80 g sugar per liter and at least 15% vol. of alcohol.

This indicates that sugar content needs as much attention as alcohol content when trying to determine possible effects of storage media on samples of forensically important fly larvae.

The largest effect detected in this study did not arise from the choice of storing media, but treatment of larvae before storage. While HWK larvae of both species stored in vodka and brandy were similar to their HWK controls in 70% ethanol, all fresh larvae were significantly different. Size differences of freshly stored samples to controls diminished over time for both species, where the effect was more pronounced in *C. vicina* larvae (Figure 3). Longer storage times might partly balance initial shrinkage effects caused by missing fixation via HWK. Interestingly, of all storage media, 70% ethanol had the largest effect on fresh larvae of *C. vicina* over all measurements, while it had the least effect of fresh *L. sericata* larvae. This result must be investigated further.

The lengths of most larvae collected and stored in alternative liquids were smaller than those of their HWK controls in 70% ethanol. This indicates a need for cautious estimations of minimal PMIs when interpreting specimen stored in alternative liquids with lower alcohol concentrations. Calculations based on size might underestimate the real development time of inspected larvae stored in media similar to those investigated here.

## 5. Conclusions

This study shows that storage of larvae in an alcoholic liquid is preferable to omitting collection at all due to lack of proper equipment. Subpar storage methods in weaker liquors than 70% ethanol did not have fatal effects on fly larvae or make them unusable for further examinations. The effect on fresh larvae was significant shrinkage compared to control samples. This can lead to an underestimation of developmental times. For larvae previously HWK on the other hand, spirits with high alcohol and low sugar contents were found to have almost negligible effects. HWK, and thus fixation of the larvae, should therefore always be aimed for when collecting fly larvae. It might help counteract effects of subpar storage media and increase comparability to existing data. It furthermore sterilizes the larvae and reduces degradation during longer storage times.

Exact documentation of treatments and storage liquids is imperative, especially in cases with makeshift methods. Casework in forensic entomology can never incorporate all influencing factors. Even less when working with external samples and/or a small amount of specimen and a lack of supplemental data. Such caveats must always be pointed out when asked specific questions in order to help an investigation. The denomination of insects developing on a human body as a “biological clock” increased expectations for the science of forensic entomology. In reality, the method cannot be as accurate as a clock, as determination of minimal PMI based on insect development must always be an estimation.

## Figures and Tables

**Table 1:** Average sizes (±SD ^1^) and size ranges of larvae in first measurement (1 day) in mm, controls are HWK larvae stored in 70% ethanol for 1 day.

Sizes	*Calliphora vicina*			*Lucilia sericata*		
	Fresh *n* = 100	HWK ^2^ *n* = 80	Control *n* = 20	Fresh *n* = 75	HWK ^2^ *n* = 60	Control *n* = 15
average	14.7 (±1.2)	15.9 (±1.2)	16.1 (±0.9)	12.7 (±0.9)	14.0 (±1.5)	15.2 (±0.7)
range	12.0–17.01	13.3–18.4	14.5–17.9	10.3–15.1	10.8–16.3	13.6–16.3

^1^ SD = standard deviation, ^2^ HWK = hot-water-killed.

**Table 2:** Average sizes of *Calliphora vicina* and *Lucilia sericata* larvae in mm, standard deviations (±SD), and number of larvae (n) measured after 1 day (1 d) and after 1 month (1 m) in alcoholic liquids, alcoholic content given in % vol., grey fields indicate controls.

Alcoholic Liquid	*Calliphora vicina*				*Lucilia sericata*			
	Fresh		HWK ^1^		Fresh		HWK ^1^	
	1 d	1 m	1 d	1 m	1 d	1 m	1 d	1 m
Ethanol 70%	12.8 * (±1.2) *n* = 20	13.8 *(±1.2)	16.3 (±0.9) *n* = 20	16.1(±0.9)	12.2 * (±0.9) *n* = 15	12.8 * (±0.8)	15.5 (±0.6) *n* = 15	15.2 (±0.7)
Vodka 37.5%	13.1 * (±1.0) *n* = 20	15.2 * (±0.8)	16.5 (±1.2) *n* = 20	16.4 (±1.1)	11.5 * (±0.6) *n* = 15	12.8 * (±0.9)	14.6 * (±0.7) *n* = 15	14.7 (±0.7)
Brandy 36%	14.2 * (±1.8) *n* = 20	15.3 * (±1.0)	16.0 (±1.2) *n* = 20	16.3 (±1.2)	12.1 * (±0.8) *n* = 15	13.2 * (±1.0)	14.5 * (±0.8) *n* = 15	15.1 (±1.0)
Jägermeister 35%	13.7 * (±1.7) *n* = 20	14.6 * (±1.1)	16.0 (±1.1) *n* = 20	15.2 * (±1.1)	11.7 * (±1.0) *n* = 15	12.3 * (±0.6)	14.1 * (±0.7) *n* = 15	12.6 * (±1.1)
Peppermint 18%	13.9 * (±1.8) *n* = 20	14.6 * (±1.3)	16.0 (±1.3) *n* = 20	15.5 (±1.3)	12.1 * (±1.0) *n* = 15	12.3 * (±0.9)	12.8 * (±1.0) *n* = 15	12.4 * (±0.9)

^1^ HWK = hot-water-killed, * statistically significant differences compared to controls (Mann–Whitney U test).

## Data Availability

The data presented in this study are available on request from the corresponding author.
